# Effects of physical activity on college students’ interpersonal competence: chain-mediated effects of self-control and prosocial interpersonal emotional management

**DOI:** 10.3389/fpsyg.2025.1649527

**Published:** 2025-08-05

**Authors:** Weidi Ni, Lili Chen

**Affiliations:** ^1^School of Exercise and Health, Shanghai University of Sport, Shanghai, China; ^2^School of Chinese Table Tennis, Shanghai University of Sport, Shanghai, China

**Keywords:** college students, physical activity, interpersonal competence, self-control, prosocial emotion, interpersonal emotional management

## Abstract

**Introduction:**

College students often struggle with developing strong interpersonal competence, which is essential for psychological well-being and social adaptation. Although the benefits of physical activity are widely recognized, the specific psychological mechanisms through which it enhances interpersonal competence remain underexplored. This study, therefore, addresses this gap by examining the mediating roles of self-control and prosocial interpersonal emotional management in the relationship between physical activity and interpersonal competence.

**Methods:**

A questionnaire survey was conducted on 847 conveniently sampled college students (Man = 382, Woman = 465, Mage = 19.22 years) in Shanghai. Pearson’s correlation analysis and chain mediation effect test was used to examine the correlation between physical activity, self-control, prosocial interpersonal emotion management, and interpersonal competence.

**Results:**

The results showed that interpersonal competence was positively correlated with physical activity, self-control, and prosocial interpersonal emotional management; physical activity was positively correlated with both self-control and prosocial interpersonal emotional management; and self-control was positively and significantly correlated with prosocial interpersonal emotion management. Higher physical activity predicts better interpersonal competence. Moreover, physical activity can indirectly affect interpersonal competence through the single mediating effects of self-control and prosocial interpersonal emotional management and the chain mediating effect of both.

**Discussion:**

The study shows that physical activity may contribute to students’ psychological well-being and supports the development of their emotional regulation and interpersonal skills. Thus, physical activity must be integrated into university education and mental health interventions to promote college students’ individual and social development.

## 1 Introduction

Interpersonal competence (IC) refers to the ability to communicate effectively, collaborate, and build relationships. It has a profound influence on multiple aspects among college students as it includes various skills such as empathy and conflict resolution (Buhrmester et al., 1988). Existing research has revealed that unqualified interpersonal relationships have emerged as major challenges to college students’ mental health, imposing a substantial psychological burden on them (Guo et al., 2024; Wu et al., 2024). In extreme cases, such barriers can disrupt their academic pursuits and daily lives (Yao et al., 2023). Studies on social support suggest that strong IC during college serves as a protective buffer against negative experiences such as loneliness (Goodfellow et al., 2022), anxiety, and depression (Gao et al., 2020), leading to better academic outcomes and achievements. Most importantly, it leads students to establish meaningful peer bonds and adapt successfully to the social demands of emerging adulthood (Klinkosz et al., 2021). Recent findings on post-pandemic IC advocate for a greater focus on developing the IC of students (Evans et al., 2024). Consequently, well-developed IC is beneficial and crucial for college students’ socialization and well-being.

IC is shaped by a complex interplay of individual and contextual factors, one of which is physical activity, widely recognized for its physical and mental health benefits. According to Havighurst’s Developmental Task Theory (Havighurst, 1948), different stages of life have different developmental tasks. People who complete the key developmental tasks expected at each stage are generally considered healthy and well-adjusted (Roisman et al., 2004). College students are in emerging adulthood which is a critical period for mastering key psychosocial skills, including self-regulation and IC, which are essential for successful adult functioning (Arnett, 2000). As college students face multiple challenges across academic, social, and career domains, developing interpersonal skills becomes a central developmental challenge. Physical activity, as a context that fosters cooperation, emotional expression, and social feedback, may play a supportive role in meeting these developmental demands at college students’ current life stage and positively shaping their long-term growth. Substantial evidence has demonstrated that appropriate physical activity is associated with positive emotional states (Wang et al., 2025), higher self-esteem, and better psychological resilience (Lin et al., 2024); further, it creates opportunities for social interaction, especially through team sports or group exercise (Novak et al., 2025). These combined benefits are crucial in establishing and sustaining healthy relationships for college students. However, little is known about whether physical activity can significantly predict IC. Therefore, it is imperative to actively explore the relationship between physical activity and college students’ IC, particularly as forming mature relationships is a central developmental task during college years. In addition, regular physical activity often demands discipline, goal-setting, and persistence, characteristics intrinsically linked to enhanced self-control, which is vital for regulating behavior and emotions in social contexts, thereby boosting IC. For example, goal-setting and task-approach motivation during exercise have been shown to positively predict dispositional self-control, which in turn supports emotional well-being and adaptive behavior (Briki, 2020).

Prosocial interpersonal emotional management (PIEM) refers to the regulation of emotions in a way that benefits both the individual and others in the social network, facilitating harmonious and supportive social interactions (Austin et al., 2018). Physical activity, especially in group settings, provides practical ground for developing PIEM, as students learn to handle social dynamics and manage interpersonal emotions effectively. Interventions that include social goal-setting and behavior management in physical activity settings have been found to improve prosocial behavior and peer interaction, particularly in children (Aadland et al., 2024). Enhanced prosocial emotional regulation through physical activity translates directly into more positive and skillful social interactions. Consequently, this study incorporates self-control and PIEM as mediating factors based on the Developmental Task Theory, aiming to reveal the complex mechanisms involved and offer practical implications for promoting college students’ interpersonal relationships and socialization.

The World Health Organization recommends at least 150 min of moderate-intensity aerobic exercise weekly for individuals aged 18–64 years to enhance their health and reduce their chronic disease risks (WHO guidelines on physical activity and sedentary behaviour, 2020). Physical activity relieves negative emotions and promotes mental health by improving social and environmental perceptions. Regular physical exercise has a positive influence on psychological resilience, which in turn enhances students’ IC. Sun and Zhang (2020) find that adolescents involved in high-intensity physical activity exhibited significantly superior IC compared with those involved in low-intensity physical activity. Additionally, physical activity effectively alleviates interpersonal stress (Liu and Sun, 2023), thereby reducing hostility and impulsivity (Li and Zhang, 2023), which is crucial for maintaining composure during social conflicts. Therefore, physical activity is strongly associated with IC among college students. Thus, we propose the following hypothesis:

*H1*: Physical activity is significantly and positively associated with IC among college students.

Self-control, defined as the capacity to regulate impulses and align behaviors with long-term objectives (Hofmann et al., 2012; Tangney et al., 2018), functions through a limited resource system analogous to muscular energy - susceptible to temporary depletion yet trainable for enhancement (Baumeister et al., 2007). Emerging evidence reveals that physical exercise serves as both an acute restorative and chronic fortifier of this self-regulatory resource, particularly when involving moderate cognitive engagement (Boat and Cooper, 2019; Zhidong et al., 2021). In collegiate populations, acute exercise interventions are found to enhance inhibitory control, a core component of self-control measured by behavioral tasks and neuroelectric assessment (Kao et al., 2017). Individuals with strong self-control demonstrate healthier relationships by managing their behaviors and thought patterns in social settings; high self-control is associated with secure attachments and relational stability (Cheung et al., 2014). Therefore, we propose the following hypothesis:

*H2*: Self-control mediates the relationship between physical activity and IC among college students.

Interpersonal emotional management (IEM) refers to the process by which people manage their emotional responses and regulate the emotions of others in social interactions (Austin et al., 2018). This ability manifests in two distinct patterns: prosocial, wherein individuals actively help others alleviate negative emotions, such as comforting a distressed friend; and strategic regulation, wherein emotion management serves personal goals, such as using flattery to gain favors. In this study, we focus on PIEM, which specifically involves the regulation of emotions in a manner that benefits both the individual and others in the social network.

Emerging evidence supports the important role of physical activity in PIEM development via neurocognitive and behavioral pathways. Neuroimaging studies reveal that sustained aerobic exercise induces structural plasticity in the anterior cingulate and medial prefrontal cortexes (Lin et al., 2020), these regions are associated with emotional monitoring and social cognition, potentially contributing to individuals’ capacity to process their own emotional states while interpreting others’ affective cues. Research links physical activity to PIEM improvements manifested as prosocial behavioral tendencies (Hui et al., 2022). As a result, individuals who engage in regular physical activity are more empathetic, adept at providing emotional support to others, and capable of resolving conflicts, building trust, and communicating effectively (Rimé et al., 2020). A recent randomized controlled trial shows that yoga-based practices enhance prosocial behaviors and promote agreeableness and peer acceptance, thereby reducing interpersonal conflict among college students (Dagar et al., 2025). Therefore, we propose the following hypothesis:

*H3*: PIEM mediates the relationship between physical activity and IC among college students.

Previous research has showed that self-control is a significant promoter of prosocial behavior, with PIEM emerging as a potential mediating factor (Wang H. et al., 2021; Li et al., 2022). From the perspective of criminology, poor self-control correlates with delinquency and criminal convictions, as individuals struggle to suppress self-interested decisions (Kundakova et al., 2022). Conceptually, self-control acts as a “brake” to suppress negative impulses, while PIEM functions as a “steering wheel” to guide emotions toward socially beneficial outcomes. For example, individuals with high self-control demonstrate greater interpersonal forgiveness even after unfair treatment (Liu and Li, 2020), likely due to their enhanced capacity to regulate dominance-related emotions (e.g., anger) and distress-related emotions (e.g., anxiety) (Paschke et al., 2016). Therefore, self-control may inhibit negative emotional escalation and promote PIEM-driven prosocial interactions. Thus, we propose the following hypothesis:

*H4*: Self-control and prosocial interpersonal emotional management chain mediate the relationship between physical activity and IC among college students.

In summary, this study aims to examine (1) the relationship between physical activity, self-control, PIEM, and IC, and (2) the chain mediating roles of self-control and PIEM in this association.

## 2 Methods

### 2.1 Participants and procedures

In September 2024, a questionnaire survey was conducted on university students in eastern China (Shanghai University of Sport, University of Shanghai for Science and Technology, Jiangxi Justice Police Vocational College, Shanghai Urban Construction Vocational College) and central China (West Anhui Health Vocational College) through the Questionnaire Star online platform using a convenience sampling method. The participants’ majors mainly include physical education, law, management, and nursing. The study was approved by the Ethics Committee of the Shanghai University of Sport (102772024RT171). All methods were performed in accordance with the Declaration of Helsinki and other relevant laws and regulations. Before the formal questionnaire survey, all university students were informed of the study’s purpose and assured of the confidentiality and exclusive research use of all personal information and responses. Informed consent was obtained from all participants and the criteria and precautions for completing the questionnaire were explained within the questionnaire.

A total of 924 questionnaires were distributed. After excluding invalid questionnaires because of trap questions, omitted answers (> 10% items) and insufficient filling time (< 2 s per items), 847 valid questionnaires were recovered, resulting in a response rate of 91.67%.

### 2.2 Measurements

#### 2.2.1 International physical activity questionnaire short form (IPAQ-SF)

The International Physical Activity Questionnaire Short Form (IPAQ-SF) was used to evaluate the physical activity level of university students. This questionnaire, introduced by Qu and Li (2004), comprises seven questions on walking, moderate physical activity, and heavy physical activity. Total energy values were calculated on the basis of the metabolic equivalents (MET-min) corresponding to each type of physical activity: walking (3.3 MET-min/min); moderate physical activity, such as carrying light loads, bicycling at a regular pace, or doubles tennis (4.0 MET-min/min); and heavy physical activity, such as heavy lifting, digging, aerobics, or fast bicycling (8.0 MET-min/min). Previous research has demonstrated that this scale is valid for assessing physical activity in samples of Chinese adolescents (Hu et al., 2015). In the present study, the total MET values of the individuals in a week were calculated by collecting the total MET values of the individuals corresponding to the different types of physical activities in the questionnaire and summing them.

#### 2.2.2 Self-control scale

The Self-Control Scale, revised by Tan and Guo (2008), was used to measure self-control. This scale is divided into five dimensions: healthy habits, entertainment abstinence, temptation resistance, work focus, and impulse control. It comprises 19 questions and is based on a 5-point Likert scale (1 “not at all,” to 5 “very much”). Higher scores indicate stronger self-control ability. In this study, Cronbach’s alpha coefficient for this scale was 0.89.

#### 2.2.3 Managing the emotions of others short form (MEOS-SF)

We used the Managing the Emotions of Others Short Form scale (Austin et al., 2018) in its Chinese adaptation (Wang Q. et al., 2021) to measure respondents’ interpersonal emotion management ability. It comprises three scales: conceal (4 items, *α* = 0.67), prosocial (10 items, α = 0.92), and non-prosocial (9 items, α = 0.86). We focused on the prosocial factor because this subscale captures behaviors such as calming, encouraging, or supporting others emotionally, which align with the theoretical focus of this study on prosocial and affiliative aspects of interpersonal competence. The conceal and non-prosocial subscales were excluded, as their content reflects manipulation, suppression, or antisocial forms of emotion regulation, which fall outside the scope of the current research focused on socially constructive emotional processes. The prosocial subscale comprises 10 items measured on a 5-point Likert scale (1 “strongly disagree” to 5 “strongly agree”). The higher one’s score, the higher their prosocial interpersonal emotion management. Cronbach’s alpha coefficient of the whole scale was 0.87.

#### 2.2.4 Interpersonal competence questionnaire

The Interpersonal Competence Questionnaire was created by Buhrmester et al. (1988), and the Chinese version was translated by Wang et al. (2006). This questionnaire contains 35 items divided into five dimensions: initiation, negative assertion, disclosure, emotional support, and conflict management. All items in the scale were rated on a 5-point Likert scale (1 “strongly disagree” to 5 “strongly agree”). Higher scores indicate higher IC ability. Cronbach’s alpha coefficient of the scale was 0.96.

### 2.3 Statistical analysis

SPSS 27.0 software is used for statistical analysis. Descriptive statistics, including means, standard deviations, and percentages, were used to characterize the participants’ demographics and the study variables. Pearson’s correlation analysis was used to examine the correlation between physical activity, self-control, PIEM, and IC. We used model 6 in Hayes’ PROCESS plug-in for SPSS (version 4.2) to explore the mediating role of self-control and PIEM in physical activity and IC (Hayes, 2017). In addition, based on the analysis of demographic variables, gender and sports specialization were found to be significantly associated with IC and therefore included as control variables in the model. The bootstrap method (sampling repeated 5,000 times) estimates a 95% confidence interval for the significance testing of mediating associations. Direct or indirect associations are considered significant when the confidence interval does not include zero.

To preclude the emergence of common method bias, some of the questions in the distributed questionnaire were reverse scored, and irrelevant questions were set to control for the effect of common method bias in the data collection process. The Harman one-factor test was employed among the primary variables. Utilizing the maximum variance method test, which is based on eigenvalue 1, we found nine factors with characteristic roots >1; the largest factor had an explained variance of 33.75%, which did not surpass the critical threshold of 40% (Podsakoff et al., 2003). This suggests that no significant common method bias was present in the data of this study.

## 3 Results

### 3.1 Descriptive statistics and correlation analysis

Table 1 shows the descriptive statistics of the participants’ demographic characteristics. Participants’ ages ranged from 17 to 27 years (mean ± SD: 19.22 ± 1.50). Among the participants, 24.8% (*n* = 210) reported sports specialization, and 75.2% (*n* = 637) were non-specialized. The sample comprised 382 (45.1%) men and 465 (54.9%) women. The results of the independent-samples t-test or Mann–Whitney U-test showed that there was a significant difference between genders regarding physical activity and IC. Specifically, males reported significantly higher physical activity than females (Male: 2640 (1,173, 5,013) MET, Female: 1386 (0, 2,776) MET, *Z* = −8.072, *p* < 0.001). Furthermore, males demonstrated significantly higher interpersonal competence than females, with a small effect size (Male: 127.05 ± 23.25, Female: 120.67 ± 19.27, Cohen’s d = 0.3, *p* < 0.001).

**Table 1:** Participants’ demographics and characteristics.

Variables	Total	Proportion	t/Z	Physical activity^a^	Self-control^b^	PIEM^b^	IC^b^
	(*n* = 847)	%	*p*	M (P_25_, P_75_)	Mean ± SD	Mean ± SD	Mean ± SD
Gender							
Male	382	45.1		2,640 (1,173, 5,013)	59.64 ± 12.17	36.13 ± 6.73	127.05 ± 23.25
Female	465	54.9		1,386 (0, 2,776)	60.38 ± 10.33	36.41 ± 6.33	120.67 ± 19.27
			Cohen’s d /Z	−8.072	−0.066	−0.043	0.3
			*p*	< 0.001	0.346	0.53	< 0.001
Sport specialization							
Yes	210	24.8		3,813 (2,553, 6,186)	60.48 ± 11.90	37.13 ± 6.82	130.63 ± 21.36
No	637	75.2		1,386 (0, 2,772)	59.90 ± 10.96	36.01 ± 6.39	121.22 ± 20.89
			Cohen’s d /Z	−11.771	0.05	0.17	0.45
			*p*	< 0.001	0.531	0.031	< 0.001

a, using Mann–Whitney U test; b, using independent samples t-test; PIEM, prosocial interpersonal emotion management; IC, interpersonal competence.

Sports specialization had significant differences in terms of physical activity, PIEM, and IC. Participants with sport specialization reported significantly higher physical activity than those without (With: 3813 (2,553, 6,186) MET, Without: 1386 (0, 2,772) MET, *Z* = −11.771, *p* < 0.001). Those with sport specialization also showed significantly higher PIEM compared to those without, with a small effect size (With: 37.13 ± 6.82, Without: 36.01 ± 6.39, Cohen’s d = 0.17, *p* < 0.05). Finally, participants with sport specialization exhibited significantly higher interpersonal competence than those without, representing a medium effect size (With: 130.63 ± 21.36, Without: 121.22 ± 20.89, Cohen’s d = 0.45, *p* < 0.001).

Table 2 shows the results of the Pearson correlation analysis, suggesting that physical activity was positively correlated with self-control (*r* = 0.09, *p* < 0.05), PIEM (*r* = 0.10, *p* < 0.01), and IC (*r* = 0.23, *p* < 0.01), which is consistent with the previous theoretical analysis. The remaining variables were two-by-two correlated with each other.

**Table 2:** Correlation between variables (*N* = 847).

	1	2	3	4
1. Physical activity	1			
2. Self-control	0.09^*^	1		
3. PIEM	0.10^**^	0.15^***^	1	
4. IC	0.23^***^	0.20^***^	0.61^***^	1

PIEM, prosocial interpersonal emotion management; IC, interpersonal competence; ^*^p < 0.05; ^**^p < 0.01; ^***^p < 0.001.

### 3.2 Chain mediation effect test

In the chain mediation effect test, gender and sports specialization were taken as the control variables, physical activity as the independent variable, IC as the dependent variable, and self-control and PIEM as the mediating variables. All variables in the model entered the regression analysis after standardized treatment.

The results of the regression analysis (Table 3) show that physical activity significantly and positively predicted college students’ IC (*β* = 0.16, *p* < 0.001). Further, after including self-control and PIEM in the regression equations, physical activity significantly and positively predicted self-control (*β* = 0.11, *p* < 0.01) and PIEM (*β* = 0.09, *p* < 0.05); self-control significantly positively predicted PIEM (*β* = 0.14, *p* < 0.001) and IC (*β* = 0.11, *p* < 0.001); and PIEM significantly positively predicted IC (*β* = 0.58, *p* < 0.001), at which point physical activity continued to be a significant predictor of IC (*β* = 0.09, *p* < 0.01).

**Table 3:** Regression analysis of variable relationships in the model (standardization).

Regression equation	Overall fit index			Significance of regression coefficient		
Result variable	Predictive variable	*R*	*R*^2^	*F*	*β*	*t*
IC	Gender	0.27	0.07	21.28	−0.17	−2.41^*^
	Sport specialization				0.26	3.07^**^
	Physical activity				0.16	4.41^***^
Self-control	Gender	0.11	0.01	3.13	0.12	1.66
	Sport specialization				−0.01	−0.17
	Physical activity				0.11	2.78^**^
PIEM	Gender	0.19	0.03	7.46	0.10	1.39
	Sport specialization				0.11	1.30
	Physical activity				0.09	2.26^*^
	Self-control				0.14	4.09^***^
IC	Gender	0.27	0.07	21.28	−0.25	−4.56^***^
	Sport specialization				0.20	2.98^**^
	Physical activity				0.09	3.20^**^
	Self-control				0.11	4.17^***^
	PIEM				0.58	22.16^***^

PIEM, prosocial interpersonal emotion management; IC, interpersonal competence; ^*^p < 0.05; ^**^p < 0.01; ^***^p < 0.001.

**Figure 1:**
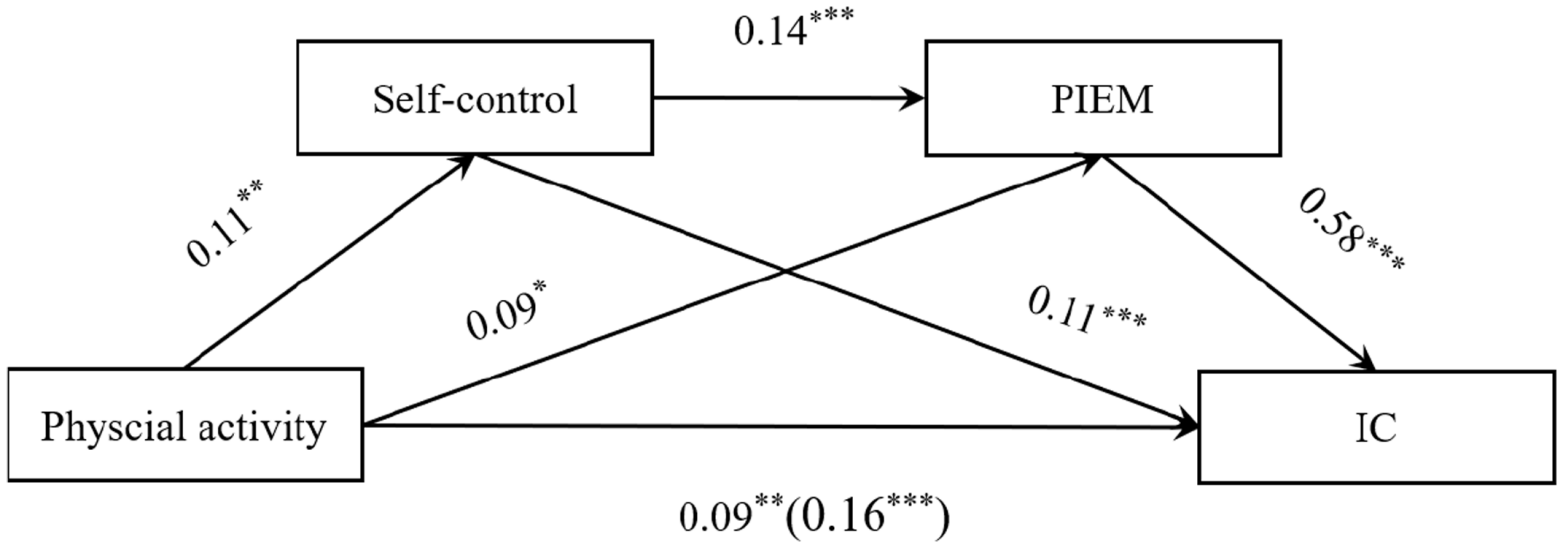
Path diagram of the chain mediation effect (standardization). ^*^p < 0.05; ^**^p < 0.01; ^***^p < 0.001; PIEM, prosocial interpersonal emotion management; IC, interpersonal competence.

The results of the mediation association size analysis show that self-control and PIEM had a significant mediation relationship between physical exercise and college students’ IC, with a total standardized direct association value of 0.09, which accounted for 56.93% of the total associations of physical exercise on the IC of college school students (0.16) (Figure 1; Table 4). The total value of the indirect associations was 0.07, including indirect associations in three pathways: the indirect association 1 of self-control (0.01); the indirect association 2 of PIEM (0.05); and the indirect association 3 of self-control and PIEM (0.01). The three indirect associations accounted for 7.15, 30.62, and 5.30% of the total associations, respectively. The 95% confidence intervals of the above indirect associations did not include zero, indicating that all three indirect associations reached a significant level.

**Table 4:** Chain-mediated model associations tests for self-control and prosocial interpersonal emotion management (standardization).

Pathway	Effect value	Bootstrap 95%CI		Proportion of relative associations
		Boot LLCI	Boot ULC	
Total associations	0.16	0.09	0.23	100%
Direct associations	0.09	0.04	0.15	56.93%
Indirect association 1	0.01	0.00	0.03	7.15%
Indirect association 2	0.05	0.01	0.09	30.62%
Indirect association 3	0.01	0.00	0.02	5.30%
Total indirect associations	0.07	0.03	0.12	43.07%

Boot LLCI and Boot ULCI refer to the standard errors and lower and upper 95% confidence intervals of the indirect associations estimated by the bias-corrected percentile Bootstrap method, respectively. Indirect association 1: physical activity → self-control →interpersonal competence; indirect association 2: physical activity → prosocial interpersonal emotion management → interpersonal competence; indirect associations 3: physical activity → self-control → prosocial interpersonal emotion management → interpersonal competence.

## 4 Discussion

This study explored the relationship between physical activity and IC among college students and its internal mechanisms. The findings reveal a significant positive correlation between physical activity and IC. Self-control and PIEM were identified as partial mediating this relationship through three pathways: the individual mediating roles of self-control and PIEM, as well as the combined chain mediation of both.

This study found a significant positive correlation between physical activity and college students’ IC, supporting Hypothesis 1 and aligning with prior findings (Sun and Zhang, 2020). Developing IC is critical for fostering friendships and cultivating supportive academic environments and future professional networks. Physically active individuals exhibit significantly higher IC and lower social interaction anxiety than do sedentary peers (Cao et al., 2023). Physical activity may contribute to IC in three ways in college students. First, physical activity often occurs in social contexts where students must collaborate with others. Here, team sports create a microcosm of social dynamics, providing opportunities to practice conflict resolution and trust cultivation, mirroring the mutual understanding required in friendships or academic collaborations, thus improving participants’ IC and harmonious interpersonal relationships. This pattern has been observed across cultures. In Japan, university students who regularly engaged in physical activity showed enhanced social problem-solving skills and interpersonal cooperation (Sone et al., 2017). In the United States, students who engaged in vigorous physical activity not only reported lower stress and better mental health, but also spent more time socializing, which in turn partially mediated the positive effects of physical activity on mental well-being (VanKim and Nelson, 2013). Second, physical exercise is thought to positively influence motivational systems within the brain, potentially increasing the drive for rewarding experiences, including social interaction, and may help reduce social anxiety (Pearce et al., 2017). While the precise neurobiological underpinnings involving systems like dopamine are complex and context-dependent, the behavioral outcome can manifest as increased social engagement. This is consistent with observations such as greater spontaneity during group exercises, potentially indicating reduced social inhibition (Tarr et al., 2015). Third, students meeting physical activity guidelines (≥ 300 min/week) have reported reduced body image concerns and higher self-esteem, particularly in boys (Liu et al., 2024). This correlates with increased social confidence and perceived attractiveness, encouraging proactive behaviors (e.g., initiating conversations) while reducing anxiety-driven avoidance.

The findings confirm the mediating effect of self-control between physical activity and IC among college students, supporting Hypothesis 2. The strength model of self-control posits that self-control relies on a limited resource that diminishes with repeated use (ego depletion) and can be enhanced through practice (Baumeister et al., 2007). Physical activity is an effective way to refill this resource pool. Students may then manage social stressors more effectively, such as resisting peer pressure and maintaining emotional composure during conflicts. Further, the shifting priorities model of self-control considers motivational and attentional processes (Fujita et al., 2025). For example, physical activity shifts one’s attention from immediate temptations to long-term goals by altering their goal valuation (Boat and Cooper, 2019). During the exercise process, individuals may overcome difficulties and persevere in completing tasks, thus cultivating self-discipline that transfers to improved impulse control in academic and social tasks. High self-control promotes the cool-headed processing of social cues, allowing individuals to prioritize their long-term relationship goals over short-term emotional gratification. Students with higher self-control are better equipped to adapt to school life as it supports behaviors such as managing their time and focusing on academic tasks, thus freeing cognitive resources for social skill development.

Our decision to focus on the prosocial subscale of the MEOS-SF to assess PIEM reflects our interest in socially constructive aspects of interpersonal competence. While concealment and manipulative strategies are also important in broader emotion regulation research, they do not align with the study’s aim of exploring positive interpersonal development through physical activity. This study found that PIEM is an essential mediating variable in the relationship between physical activity and IC among college students, supporting Hypothesis 3. This aligns with previous studies suggesting that social–emotional competency is often a crucial factor in the relationships between physical activity and prosocial interpersonal outcomes (Hill et al., 2024). Emotions often significantly influence behaviors, and such neural adaptations enhance emotion regulation capacity, enabling individuals to replace hostile reactions with prosocial strategies, such as reacting with empathy instead of hostility. Physical activity significantly improves positive emotions in adolescents, which in turn may contribute to their IC. According to the self-determination theory, when students make progress or achieve their goals through exercise, their self-confidence and self-efficacy are enhanced. This positive self-perception will enhance their prosociality and extend into their social lives, making them more willing to take the initiative to interact with others and enabling them to be more confident in social interactions. Notably, our model advances this theory by introducing PIEM as an interpersonal-level mediator. This addition helps to capture the cultural dimensions of emotional management. Cross-cultural findings highlighted the differences in emotional regulation strategies (Tamir et al., 2024). Our samples were collected from universities in East Asia, where collectivist cultural norms may shape the expression and interpretation of interpersonal behaviors. In such contexts, prosocial emotion management may be more strongly influenced by social harmony, group conformity, and relational obligation, compared to more individualistic societies where emotional expression tends to be more autonomous and self-directed. Therefore, the findings may reflect culturally specific patterns of PIEM that are not entirely generalizable to Western or individualistic populations.

Finally, this study found that self-control and PIEM were chain mediators in the relationship between physical activity and IC among college students, supporting Hypothesis 4. Prior research has found that self-control during adolescence predicted prosocial tendencies into adulthood, indicating that self-control helps adolescents manage emotions and develop prosociality (Alessandri et al., 2014). Further, adolescents high in self-control reported more prosocial behavior (Nie et al., 2016), and this pattern is replicated in our findings, wherein self-control positively predicted PIEM, indicating that students with high self-control care more about others’ welfare in social interactions. Being prosocial is known to be promoted by most cultures, social values, and religions, but certain situations indicate a dilemma between “individualism” and “altruism,” in which being prosocial often requires self-sacrifice. However, a lack of self-control may result in selfishness and undermine prosociality (Joosten et al., 2015), as self-control restrains one’s response to align with social morality, virtues, and social norms. Therefore, college students can increase their self-control through physical activity based on the strength model of self-control. Adequate self-control resources enable individuals to recognize their emotional fluctuations and others’ social cues. This prompts them to evaluate their immediate behaviors against ideals and social expectations and consequently suppress their impulsive negative emotions such as anger or anxiety.

Although the current study is primarily behavioral and correlational in design, previous neurobiological research helps contextualize our findings. Physical activity has been shown to influence brain regions involved in emotional regulation and self-control, particularly the prefrontal cortex (PFC) and amygdala. The PFC, especially the anterior and dorsolateral regions, is central to self-regulation and impulse control, both of which are foundational for managing social–emotional behavior (Kaldewaij et al., 2021). Exercise-induced changes in prefrontal activity have been associated with improved emotional control and resilience. Moreover, functional connectivity between the PFC and the amygdala has been shown to predict individual differences in successful emotion regulation (Morawetz et al., 2017). Physical activity appears to modulate this circuitry, potentially dampening amygdala reactivity through enhanced prefrontal engagement (Lohaus et al., 2024). Although our study does not directly measure brain function, these findings support a plausible neurobiological pathway through which physical activity could influence the psychosocial variables we examined.

### 4.1 Implications

The findings offer several important implications. First, we identify the potential for using physical activity as a tool to support students’ emotional regulation and social relationships, which are vital for their academic and social transitions, thereby helping them achieve the core developmental goals of young adulthood. Second, parents and universities should actively promote group-based physical activities, such as team sports, dance classes, or cooperative games, that provide accessible, inclusive settings where students can build social connections and develop prosocial emotional management skills relevant for successful transitions into adulthood. Additionally, campus recreation programs, including intramural sports leagues, group fitness classes, outdoor adventure trips, and wellness workshops, have been shown to reduce stress and anxiety, foster a sense of belonging, and improve overall life satisfaction among college students (Lifschutz, 2019; Abdeahad and Mock, 2023). Physical education should not be confined to physical fitness training; instead, it could incorporate social–emotional learning components, such as empathy-building exercises, peer cooperation, and emotion regulation strategies, which may offer long-term benefits for students’ career development and social integration. Finally, rather than treating physical activity, self-control, and prosocial emotions as isolated factors, educators should emphasize their interactive and reinforcing effects, integrating them into holistic educational models to promote both personal and interpersonal growth in college students.

### 4.2 Limitations

This study has several limitations. First, data were collected through self-report questionnaires which may have been influenced by social desirability bias or recall errors. Future research should adopt objective behavioral indicators. For instance, employing wearable devices like accelerometers would enable objective tracking of physical activity. Second, this cross-sectional study examined only associations, thus limiting causal inference. Longitudinal studies would provide a better understanding of the long-term effects of physical activity on IEM and IC. Third, cultural differences in prosocial norms may limit the generalizability of our findings. As most references and data are from Chinese populations, future research directions should address the replicability of findings in Western samples with individualistic emotional regulation strategies. Finally, our analysis focused on total physical activity MET values, self-control, PIEM, and total IC scores. The correlations among intensity and types of physical activity, the sub-dimensions of IC, non-prosocial aspects, and personality traits remain unexplored.

## 5 Conclusion

Our study is the first to demonstrate that physical activity, self-control, and PIEM significantly predict college students’ IC within the framework of the Developmental Task Theory, where self-control and PIEM act as chain mediators. The findings suggest that physical activity enhances students’ physical well-being and critically supports the development of their emotional regulation and interpersonal skills. Therefore, integrating physical activity into university education and mental health interventions is imperative to promote college students’ individual and social development.

## Data Availability

The original contributions presented in the study are included in the article/supplementary material, further inquiries can be directed to the corresponding author.
